# Abnormalities of Hippocampal Subfields in Individuals With Acute Carbon Monoxide Poisoning

**DOI:** 10.1111/cns.70482

**Published:** 2025-06-23

**Authors:** Mengyue Tang, Ting Li, Yan Deng, Yifan Ji, Siyue Wang, Nian Liu, Xiaohua Huang, Xiaoming Zhang

**Affiliations:** ^1^ Sichuan Key Laboratory of Medical Imaging, Department of Radiology Nanchong Sichuan PR China

**Keywords:** carbon monoxide poisoning, CO, delayed encephalopathy, hippocampus, subfield

## Abstract

**Objective:**

To investigate alterations in hippocampal subfields in patients with acute carbon monoxide poisoning (ACMP) and explore their relationship with neurocognitive function.

**Materials and Methods:**

Forty‐seven ACMP patients and 29 age‐ and sex‐matched healthy controls (HCs) were recruited. All ACMP patients underwent carboxyhemoglobin (COHb) assessment at admission and acquired MRI scans within 3 days post‐exposure. Cognitive functions were assessed using the mini‐mental state examination (MMSE) and Montreal Cognitive Assessment (MoCA), and activities of daily living were evaluated using the Functional Independence Measure (FIM) and Barthel Index (BI). Differences in hippocampal volume between groups were analyzed using Analysis of Covariance (ANCOVA), and correlations with cognitive and functional scores were evaluated.

**Results:**

After follow‐up, 27.66% (13/47) of ACMP patients developed Delayed Encephalopathy After Carbon Monoxide Poisoning (DEACMP). The COHb concentration was significantly higher in the DEACMP group (median 17.70% vs. 11.95%, *z* = −2.225, *p* = 0.026) compared to the Recovery group. The cognitive function scores, delayed memory‐related sub‐items scores derived from cognitive assessments, and activities of daily living scores in the DEACMP group were lower than those in the Recovery group (all *p* < 0.05). The ACMP group showed significant volume reduction in the bilateral whole hippocampus, cornu ammonis (CA) cornu ammonis 3, CA4, GC.ML.DG, Moleculat_layer, and right subiculum compared to HCs. The right subiculum and right CA4 volumes were smaller in the DEACMP group than in the Recovery group. The ROC curve analysis indicated that the combination of COHb concentration, MoCA, and FIM scores had good predictive value for DEACMP(the area under the ROC curve = 0.887, *p* < 0001). Correlation analysis showed that MoCA‐delayed recall was positively associated with the volume of the left CA1 subfield (*r* = 0.357, *p* = 0.020), and MMSE‐delayed recall was positively associated with the volume of the left presubiculum (*r* = 0.323, *p* = 0.037).

**Conclusion:**

This study is the first to report specific hippocampal subfield alterations in ACMP patients, suggesting their potential as non‐invasive markers of hippocampal injury. The hippocampal subfields may contribute to the development of DEACMP by modulating cognitive processes. These findings may improve understanding of the neurological impact of hypoxic injuries in human subject research.

## Graphical abstract

This study investigated the relationship between hippocampal subfield volumes and cognitive impairments in patients with ACMP. We found significant volume reductions in the bilateral CA3, CA4, GC.ML.DG, Moleculat_layer, and right subiculum in ACMP patients. Among ACMP patients, patients who developed DEACMP had smaller volumes in the right CA4 and right subiculum. Specific hippocampal subfields may be involved in the development of DEACMP by potentially modulating cognitive processes.

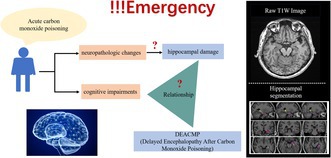

## 1 Introduction

Carbon monoxide (CO) poisoning is one of the most common causes of accidental poisoning worldwide [1, 2]. It is distinguished into acute poisoning resulting from brief exposure to elevated CO concentrations and chronic poisoning stemming from prolonged or recurrent exposure to lower concentrations. The impact of Acute Carbon Monoxide Poisoning (ACMP) transcends immediate symptoms, potentially yielding long‐term neurological repercussions. The most significant consequences are brain injury and mortality, and approximately 40% of those who survive the initial incident develop Delayed Encephalopathy After Carbon Monoxide Poisoning (DEACMP) [3].

ACMP could result in neuropathologic changes and cognitive impairments due to anoxia and other related biochemical mechanisms [4]. CO poisoning could cause neurons to undergo apoptosis, necrosis, and oxidative stress within a short period of time (several hours or days) [5, 6]. While the acute effects of CO poisoning, such as headaches, dizziness, and loss of consciousness, have been extensively documented, the enduring impact on specific brain regions remains an ongoing area of investigation. The hippocampus, a region crucial for regulating cognitive [7, 8], is known to be vulnerable to the damage of hypoxia, ischemia, or encephalitis, etc. [9, 10] Previous animal studies indicate post‐traumatic hypoxia could exacerbate hippocampal neuronal cell death [11]. The autopsy results of carbon monoxide poisoning‐induced brain damage indicate that approximately half of the cases have suffered hippocampal injuries [12]. Chen et al. [13] found that patients after carbon monoxide (CO) poisoning exhibited abnormalities in hippocampal morphological indicators in the chronic phase.

Although previous studies had indicated the association between CO exposure and hippocampal injury [14], there has not been a detailed investigation into the specific changes within the hippocampal subfields. As we know, the hippocampus is a heterogeneous structure that can be subdivided into several subfields, and the different subfields have distinct functions [15]; for example, different hippocampal subfields are involved in a variety of cognitive processing [16, 17, 18].

At present, there is a paucity of research elucidating the specific changes within the hippocampal subfields following CO exposure, and the relationship between acute hippocampal injury in ACMP and its association with cognitive function remains poorly understood. Hence, our study aims to investigate hippocampal alterations in its subfields level in ACMP and explore the relationship between hippocampal subfields volume changes and cognition injury, seeking to identify early biomarkers for DEACMP. Our findings may not only help clarify the pattern of hippocampal subfield changes after ACMP but also may potentially provide new insights into the neurological sequelae of CO exposure.

## 2 Methods

### 2.1 Subjects

This prospective study was approved by the local Research Ethics Committee, and informed written consent was obtained from all participants prior to study participation. A total of 47 patients with ACMP and 29 age‐ and sex‐matched healthy controls (HCs) were recruited. Inclusion Criteria: (1) The diagnostic criteria of GBZ23‐2002 in acute carbon monoxide poisoning; (2) All participants were right‐handed and native Han Chinese; (3) MRI scan within 3 days after CO exposure. Exclusion Criteria: (1) age < 18 years; (2) patients with a history of encephalitis, traumatic brain injury, vascular diseases, metabolic disorders, epilepsy, psychiatric disorders, or other severe neurological symptoms and signs; (3) patients with severe systemic diseases; (4) patients with alcohol or drug dependence; (5) patients with delayed‐onset cerebral diseases.

After a follow‐up of more than 3 months, if ACMP patients experienced a false recovery period of 2–60 days after the recovery of consciousness of acute CO poisoning, and then reappear with the following clinical manifestations: consciousness disorders, abnormal mental behavior, pyramidal system nerve damage, extrapyramidal system nerve dysfunction, focal functional disorders of the cerebral cortex, and brain dysfunction mainly involving cranial and peripheral nerves, they are classified as the DEACMP group [19, 20]. While the ACMP patients do not exhibit the aforementioned symptoms after waking up from a coma, they classified into the Recovery Group.

HCs Group Inclusion Criteria: (1) No systemic diseases and neurological symptoms or signs; (2) Routine head MRI shows no apparent structural abnormalities; (3) Without psychiatric disorders, including alcohol or drug dependence; (4) Right‐handed.

### 2.2 Clinical Assessment

Collect the carboxyhemoglobin (COHb) concentration indicators at the time of patient admission and evaluate these patient's status. Cognitive function assessments were conducted on all enrolled subjects using the mini‐mental state examination (MMSE) and Montreal Cognitive Assessment (MoCA). Due to the well‐established relationship between the hippocampus and memory [21], we specifically focused on the memory‐related dimensions within the MMSE and the MoCA to evaluate memory function in all enrolled subjects. Additionally, Activities of daily living (ADL) assessments were conducted on all enrolled subjects using Functional Independence Measurement (FIM) and Barthel index (BI).

### 2.3 MRI Data Acquisition and Processing

All cases underwent an MRI scan within 3 days of poisoning. MRI data were acquired on a 3.0 T MRI system (GE, Discovery MR750, United States) and with a standard 32‐channel head coil. A 3D‐T1 BRAVO was used; the parameters were as follows: repetition time (TR) = 8.2 ms, echo time (TE) = 3.2 ms, flip angle = 12°, field of view (FOV) = 240 mm × 240 mm, matrix = 256 × 256, 140 axial slices with 1.0 mm thickness, and no gap. AX T2 FLAIR and 3D PCASL sequence images were also acquired, with exclusion criteria applied to subjects with pre‐existing intracranial lesions.

**FIGURE 1:**
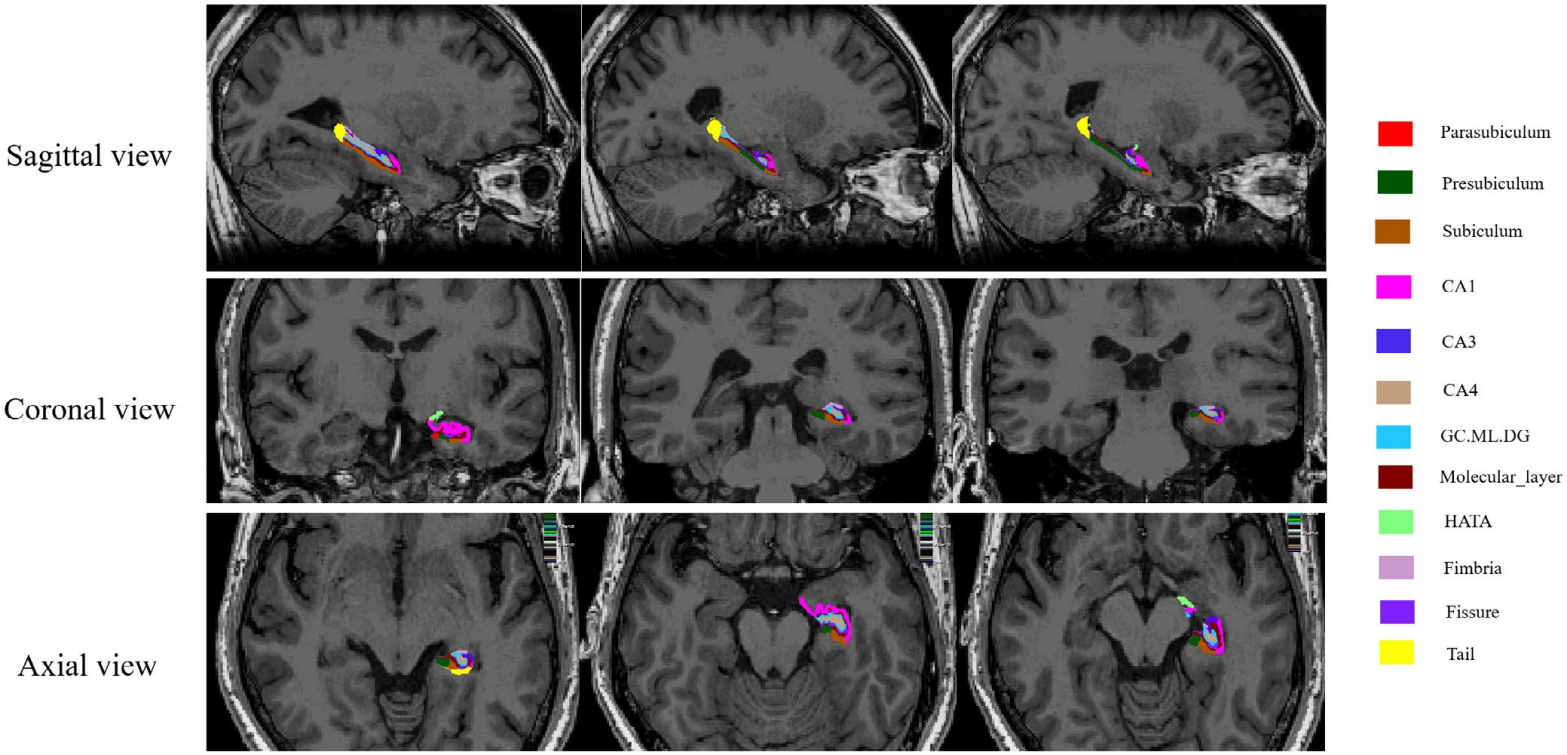
Hippocampus subfields segmentation using FreeSurfer overlaid on the T1‐weighted image. CA, cornu ammonis; GC.ML.DG, Granule cell and molecular layer of the dentate gyrus; HATA, Hippocampus‐amygdala transition area.

Image processing for the 3D T1 images was performed using FreeSurfer version 7.1.1. This segmented both hippocampus of each subject into 12 subfields: parasubiculum, presubiculum, subiculum, cornu ammonis (CA) 1, CA3, CA4, Granule cell and molecular layer of the dentate gyrus (GC.ML.DG), molecular_layer, Hippocampus‐amygdala transition area (HATA), fimbria, hippocampal fissure and tail. Additionally, intracranial volume (ICV) was extracted. Figure 1 showed hippocampus subfields segmentation using FreeSurfer overlaid on the T1‐weighted image.

### 2.4 Statistical Analysis

All continuous variables were presented as the mean or median. The Shapiro–Wilk test was used to assess the normality of the distribution of continuous variables. For variables that followed a normal distribution, analysis of variance (ANOVA) or analysis of covariance (ANCOVA) was applied. For variables that did not follow a normal distribution, the Mann–Whitney *U* test was used. Demographic characteristics and assessment scale scores were compared using ANOVA, Mann–Whitney nonparametric *U*‐tests, or Chi‐Squared tests. ANCOVA was used to compare hippocampal volumetric measures among groups, and post hoc analysis was performed. *p* values were corrected for multiple comparisons using false discovery rate (FDR). The correlation between the clinical parameters and hippocampal subfield volumes was investigated using partial correlation analysis corrected for sex, age, education level, intracranial volume, and COHb concentration. Receiver operating characteristic (ROC) curve analysis was used to evaluate the predictive value of hippocampal volume parameters and clinical indicators for DEACMP. Statistical analyses were performed using commercially available software (SPSS Inc., Chicago, IL, Ver. 23.0), and a two‐tailed *p* < 0.05 was considered significant.

## 3 Results

### 3.1 Demographics

The demographic characteristics of these subject groups are presented in Table 1. There were no significant differences in age, sex distribution and education level between the ACMP group and HCs. After a follow‐up of more than 3 months, 27.66% (13/47) ACMP patients developed DEACMP. The median COHb concentration among ACMP patients was 14.20% (range: 1.20%–44.80%), and the median COHb concentration was significantly higher in the DEACMP group [17.70% (range: 6.60%–38.00%)] compared to the Recovery group [11.95% (range: 1.20%–44.80%)], with a statistically significant difference (*z* = −2.225, *p* = 0.026). The cognitive function scores, memory‐related sub‐items scores and activities of daily living scores in the DEACMP group were lower than those in the recovery group (all *p* < 0.05 except for MMSE‐ Immediate recall scores, details as shown in Table 1).

**TABLE 1:** Demographic and clinical characteristics of participants.

	ACMP	DEACMP	RG	HCs	ACMP vs. HCs		DEACMP vs. RG	
					*t*/*x* ^2^	*p*	*t*/*x* ^2^/*Z*	*p*
*N*	47	13	34	29	—	—	—	—
Age	51.51 ± 16.46	55.46 ± 16.42	50.00 ± 16.46	45.00 ± 13.64	1.785	0.078	−1.02	0.319
F/M	31/16	6/7	25/9	14/15	2.322	0.128	2.038	0.153
Education	7.17 ± 3.835	7.32 ± 3.89	6.77 ± 3.81	8.03 ± 4.26	−0.915	0.363	0.443	0.663
COHb	14.20 (1.20,44.80)	17.70 (6.60,38.00)	11.95 (1.20,44.80)	—	—	—	−2.225	0.026
MoCA, median (range)	22 (4,30)	19 (4,29)	23 (9,30)	—	—	—	−2.421	0.015
MOCA‐delayed recall, median (range)	3 (0,5)	2 (0,5)	3 (0,5)	—	—	—	−2.109	0.035
MMSE, median (range)	25 (3,30)	22 (3,29)	26.50 (8,30)	—	—	—	−2.290	0.022
MMSE‐immediate recall, median (range)	2 (1,3)	2 (1,3)	2 (1,3)	—	—	—	−1.915	0.055
MMSE‐delayed recall, median (range)	2 (0,3)	1 (0,3)	2 (1,3)	—	—	—	−3.286	0.001
ADL median (range)	100 (15,100)	100 (15,100)	100 (90,100)	—	—	—	−3.083	0.002
FIM median (range)	120 (22,125)	110 (22,124)	120.5 (100,125)	—	—	—	−3.177	0.001

Abbreviations: ACMP, acute carbon monoxide poisoning; ADL, activities of daily living; COHb, carboxyhemoglobin; DEACMP, delayed encephalopathy after carbon monoxide poisoning; FIM, functional independence measurement; MMSE, mini‐mental state examination; MoCA, Montreal Cognitive Assessment; RG, recovery group.

### 3.2 Comparison of Hippocampal Subfield Volume Between the ACMP Group and HCs

**FIGURE 2:**
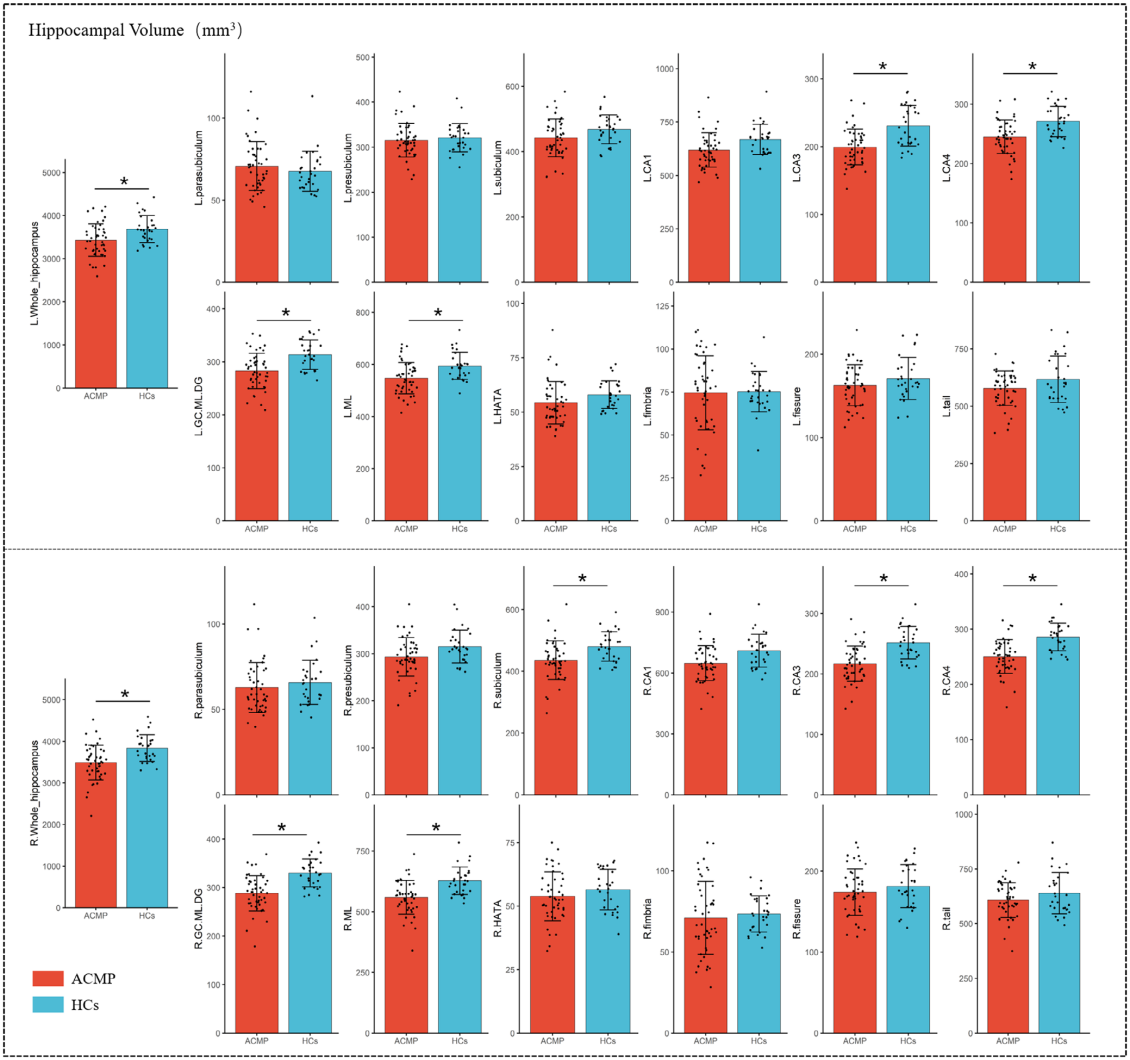
Comparison of hippocampal subfields volume between the ACMP group and HCs. *indicates a statistically significant difference after FDR correction. (*p*.FDR < 0.05). ACMP, acute carbon monoxide poisoning; HCs, healthy controls; CA, cornu ammonis; GC.ML.DG, Granule cell and molecular layer of the dentate gyrus; ML, molecular _layer; HATA, Hippocampus‐amygdala transition area.

**FIGURE 3:**
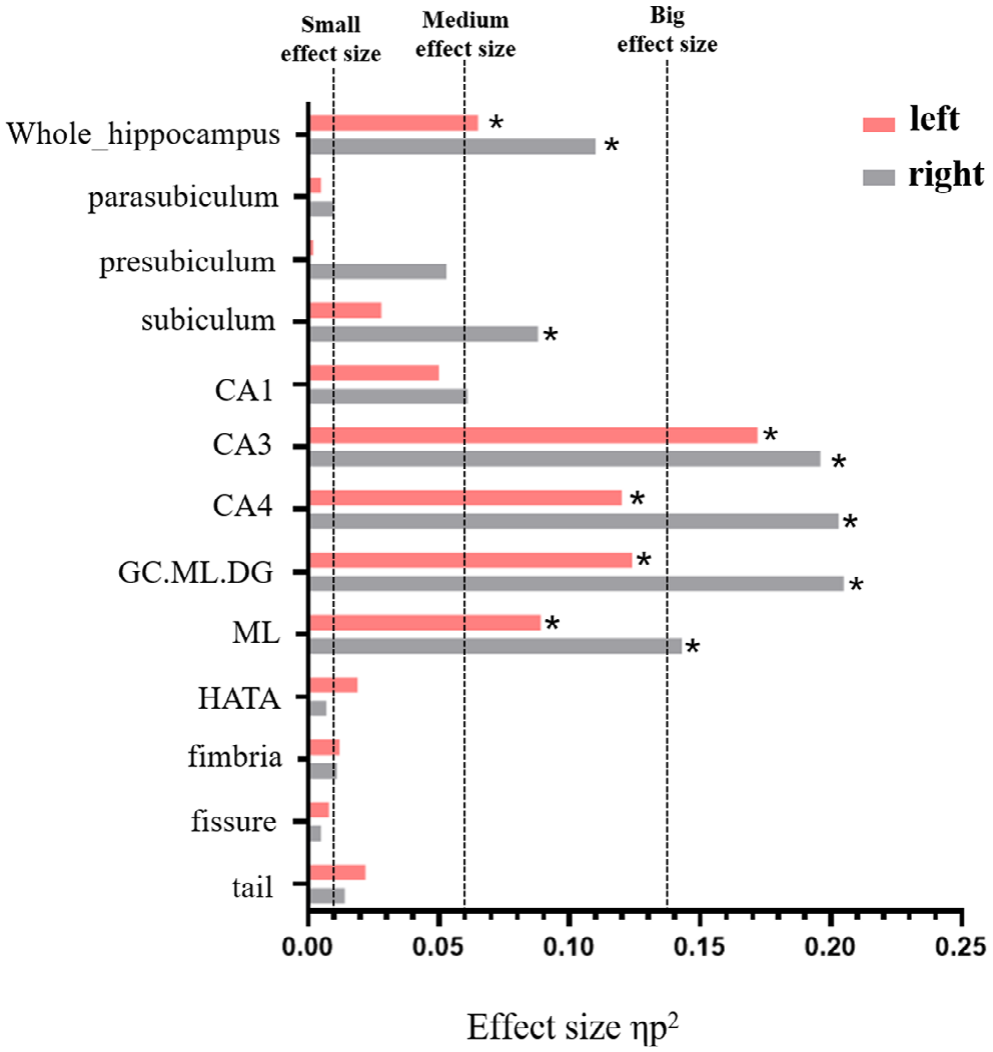
Effect size of of group differences in bilateral whole hippocampus and hippocampal subfields between the ACMP and HCs groups. ACMP, acute carbon monoxide poisoning; HCs, healthy controls; CA, cornu ammonis; GC.ML.DG, Granule cell and molecular layer of the dentate gyrus; ML, molecular _layer; HATA, Hippocampus‐amygdala transition area. * indicates a statistically significant difference after FDR correction. (*p*.FDR < 0.05).

The bilateral whole hippocampus showed significant decreased volume in the ACMP group compared to HCs. In terms of the subfields level, the significant decrease was detected in the bilateral CA3, CA4, GC.ML.DG, Moleculat_layer, and right subiculum (Table 2, Figures 2 and 3).

**TABLE 2:** Comparison of hippocampal subfields volume.

	ACMP	RG	DEACMP	HCs	*F*	*p*	*F*	*p*	*p*	*p*	*p*
	*N* = 47	*N* = 34	*N* = 13	*N* = 29	ACMP VS. HCs		RG vs. DEACMP vs. HCs		RG vs. DEACMP	RG vs. HCs	DEACMP vs. HCs
L.Whole_hippocampus	3431.13 ± 376.7	3442.15 ± 405.1	3402.34 ± 302.1	3686.88 ± 313.8	5.945	0.017*	3.611	0.032*	0.203	0.057	0.025*
L.parasubiculum	70.79 ± 14.9	71.89 ± 16.3	67.94 ± 10.3	67.64 ± 12.2	0.395	0.532	1.419	0.249	—	—	—
L.presubiculum	315.49 ± 37.3	319.79 ± 42.3	304.24 ± 14.1	320.81 ± 31.4	0.194	0.661	2.216	0.117	—	—	—
L.subiculum	442.28 ± 57.6	445.01 ± 62.5	435.12 ± 43.6	468.21 ± 43.8	2.366	0.129	1.856	0.164	—	—	—
L.CA1	620.07 ± 80.4	622.61 ± 88.3	613.43 ± 57.3	669.15 ± 70.8	4.396	0.04	2.91	0.061	—	—	—
L.CA3	199.51 ± 26.5	196.26 ± 25.2	208.01 ± 28.7	230.74 ± 29.9	17.693	< 0.001*	9.167	< 0.001*	0.387	< 0.001*	0.062
L.CA4	245.26 ± 28.3	244.28 ± 29.4	247.84 ± 26	270.99 ± 25.5	11.925	0.001*	5.894	0.004*	0.728	0.002*	0.033*
L.GC.ML.DG	282.83 ± 33.3	282.5 ± 34.2	283.68 ± 32	313.63 ± 27.8	12.825	0.001*	6.475	0.003*	0.494	0.002*	0.015*
L.molecular_layer	547.7 ± 60.6	549.8 ± 65.5	542.21 ± 47.3	595.01 ± 52.1	8.267	0.005*	4.775	0.011*	0.201	0.024*	0.012*
L.HATA	54.34 ± 9.8	54.07 ± 9.2	55.04 ± 11.5	58.04 ± 6.3	1.451	0.232	0.715	0.493	—	—	—
L.fimbria	74.53 ± 21.6	77.5 ± 20.2	66.78 ± 23.9	75.23 ± 11.7	1.368	0.246	2.967	0.058	—	—	—
L.fissure	162.87 ± 24.5	164.57 ± 25.6	158.42 ± 21.8	170.93 ± 25.2	0.604	0.44	0.926	0.401	—	—	—
L.tail	578.33 ± 75	578.44 ± 76.2	578.05 ± 74.8	617.41 ± 101.5	1.719	0.194	0.923	0.402	—	—	—
R.Whole_hippocampus	3488.24 ± 418.4	3516.31 ± 391.4	3414.84 ± 491.8	3837.04 ± 19.4	10.999	0.001*	7.624	0.001*	0.055	0.005*	0.002*
R.parasubiculum	62.81 ± 14.6	63.17 ± 16	61.89 ± 10.8	65.82 ± 12.9	0.871	0.354	1.098	0.339	—	—	—
R.presubiculum	293.5 ± 40.6	296 ± 41.1	286.94 ± 40.3	315.51 ± 34.8	4.418	0.039	3.47	0.037*	0.122	0.106	0.024*
R.subiculum	435.95 ± 62.3	440.63 ± 63.9	423.71 ± 58.6	480.46 ± 47.4	7.931	0.006*	5.977	0.004*	0.042*	0.032*	0.003*
R.CA1	648.2 ± 85.9	654.12 ± 81.5	632.71 ± 98.4	710.2 ± 81.5	5.543	0.021	4.251	0.018*	0.095	0.06	0.02*
R.CA3	216.95 ± 29.2	218.9 ± 24.2	211.84 ± 40.3	251.66 ± 27	20.653	< 0.001*	11.661	< 0.001*	0.137	< 0.001*	0.001*
R.CA4	250.59 ± 30.6	253.48 ± 26.4	243.03 ± 39.8	285.88 ± 25.1	21.945	< 0.001*	13.582	< 0.001*	0.04*	< 0.001*	< 0.001*
R.GC.ML.DG	288.13 ± 36.4	291.27 ± 31.4	279.91 ± 47.5	330.22 ± 28.9	23.495	< 0.001*	14.456	< 0.001*	0.051	< 0.001*	< 0.001*
R.molecular_layer	559.45 ± 69.8	564.78 ± 64.1	545.53 ± 84	628.11 ± 56.8	14.802	<0.001*	9.441	< 0.001*	0.056	0.001*	0.001*
R.HATA	53.86 ± 9.7	54.06 ± 9	53.34 ± 11.6	56.5 ± 8	0.516	0.475	0.434	0.65	—	—	—
R.fissure	174.21 ± 28.7	176.35 ± 27.1	168.61 ± 33.2	181.45 ± 26.5	0.865	0.356	1.21	0.305	—	—	—
R.fimbria	71.04 ± 22.4	70.53 ± 21.2	72.38 ± 26.4	73.46 ± 11.2	0.456	0.502	0.226	0.798	—	—	—
R.tail	607.76 ± 79.5	609.38 ± 72.2	603.55 ± 99.4	639.23 ± 94.8	1.078	0.303	0.949	0.392	—	—	—

Abbreviations: ACMP, acute carbon monoxide poisoning; DEACMP, delayed encephalopathy after carbon monoxide poisoning; HCs, healthy controls; RG, recovery group; CA, cornu ammonis; GC.ML.DG, Granule cell and molecular layer of the dentate gyrus; HATA, Hippocampus‐amygdala transition area.

* Indicates a statistically significant difference after FDR correction (*p*.FDR < 0.05).

### 3.3 Comparison of Hippocampal Subfield Volume Among the DEACMP, Recovery Group and HCs

**FIGURE 4:**
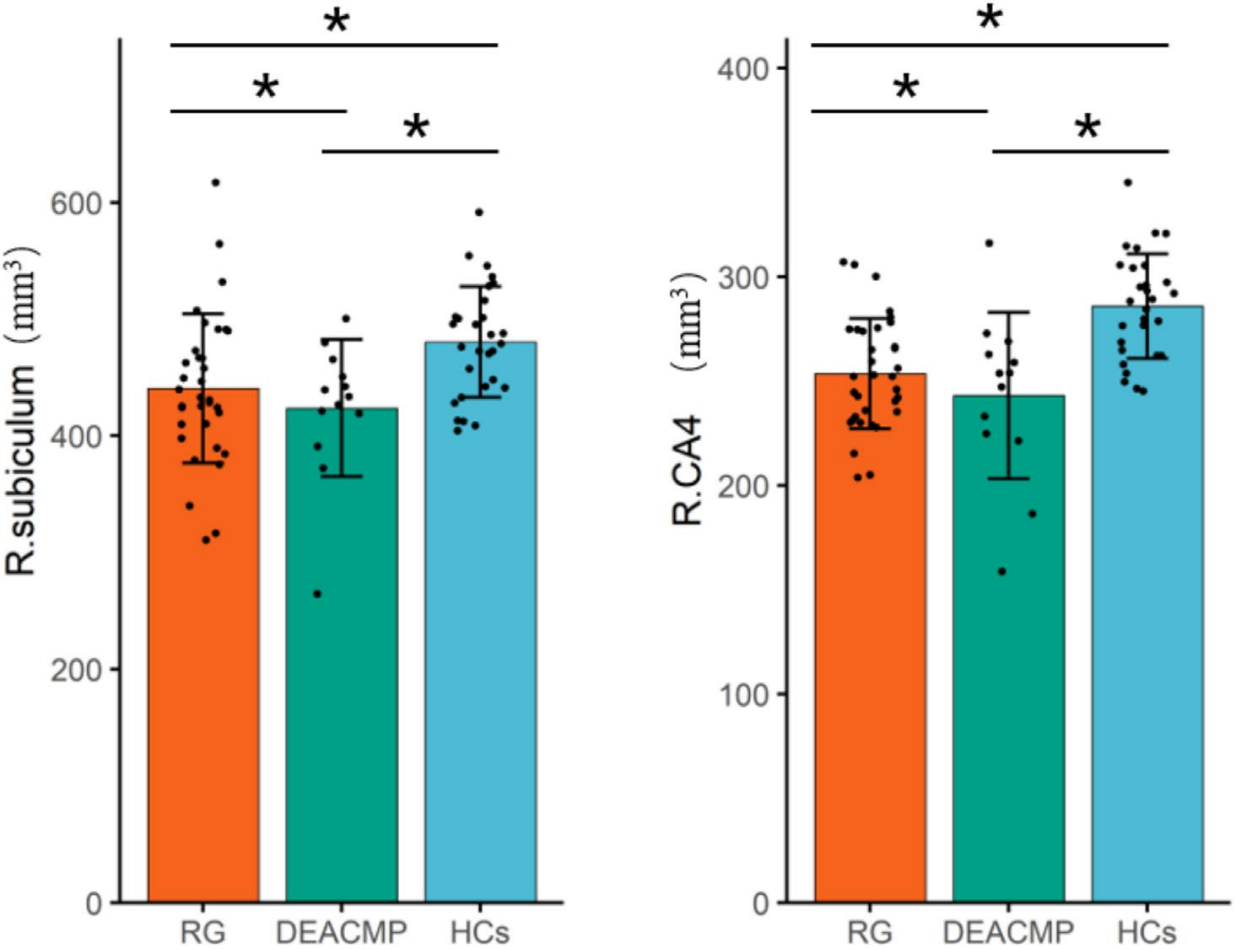
Comparison of right subiculum and CA4 volume among the RG, DEACMP, and HCs. RG, Recovery group; DEACMP, Delayed Encephalopathy After Carbon Monoxide Poisoning; HCs, healthy controls. *indicates a statistically significant difference after FDR correction. (*p*.FDR < 0.05).

The bilateral whole hippocampus showed significant difference among these three groups. In terms of the subfields level, the bilateral CA3, CA4, GC.ML.DG, Moleculat_layer, right subiculum and presubiculum also showed significant difference among these three groups. Subsequent post hoc test revealed that statistically significant differences between two groups primarily were observed in the patient group (including the DEACMP group and recovery group) and the HCs. The statistically significant differences between the DEACMP group and recovery group were only detected in the right subiculum and right CA4, with smaller volume in the DEACMP group (Figure 4 and Figures S1 and S2). The analysis details were provided in the Table 2.

### 3.4 Prediction Analysis for DEACMP

**FIGURE 5:**
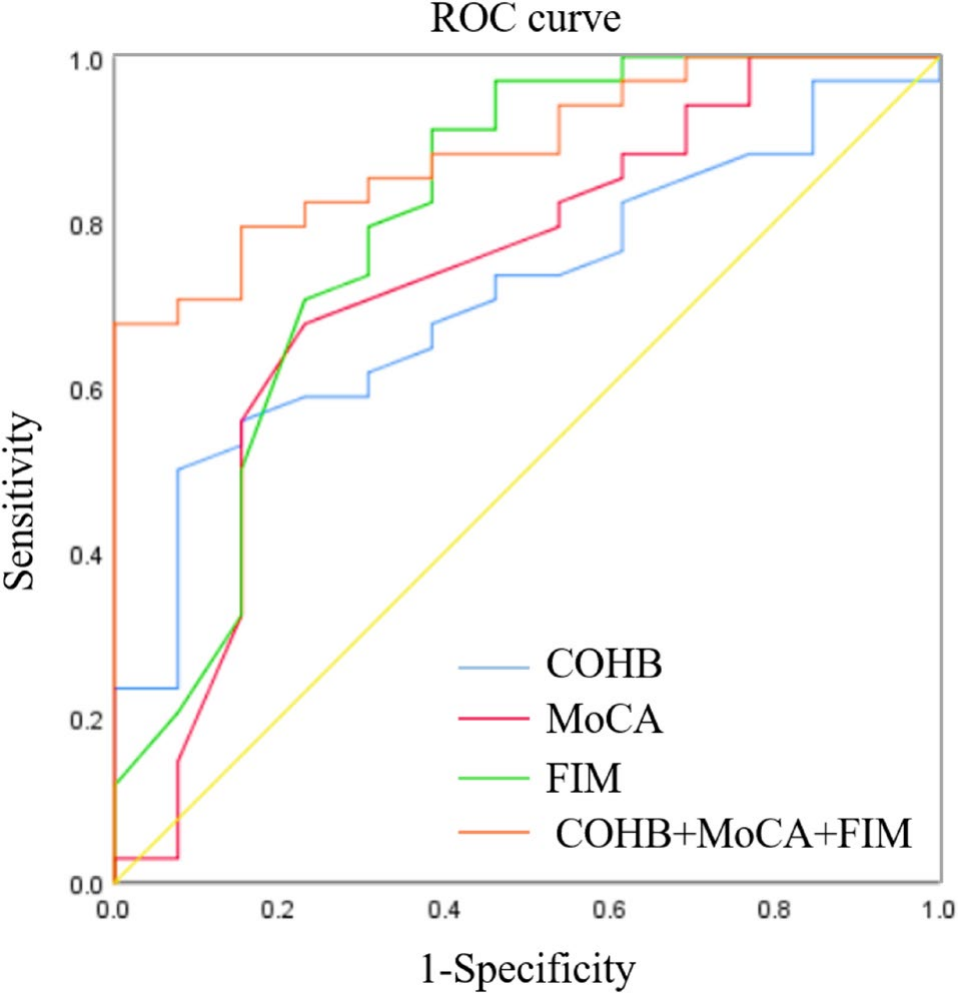
ROC curve analysis to evaluate the prediction value for DEACMP. It showed that the COHb concentration, MoCA score and FIM scores each significantly predict DEACMP, and combining these three indicators enhanced the predictive performance. ROC, Receiver operating characteristic; DEACMP, Delayed Encephalopathy After Carbon Monoxide Poisoning; COHb, carboxyhemoglobin; MoCA, Montreal Cognitive Assessment; FIM, Functional Independence Measurement.

Based on our above results, the DEACMP group had smaller right subiculum and CA4 volume, higher COHB concentration, and lower clinical relevance scores compared to the recovery group. We used ROC analysis to evaluate the predictive value for DEACMP. We found that the COHB concentration, MoCA, MoCA‐delayed recall, MMSE, MMSE‐delayed recall, ADL, and FIM scores significantly predict DEACMP, respectively (Table S1 and Figure S3), not in the hippocampal volume parameters. Moreover, multicollinearity between variables was tested using tolerance and variance inflation factor, and finally, these COHB concentration, MoCA, and FIM were included for joint analysis to predict DEACMP (Figure 5). The results showed that the area under the ROC curve (AUC) for the combination of the three indicators was 0.887(*p* < 0001).

### 3.5 The Relationship Between Hippocampal Volume and Cognitive Function in ACMP

We defined patients with a MoCA score below 26 or an MMSE score below 27 as having cognitive impairment. 57.47% (27/47) ACMP patients had cognitive impairment. The group comparison showed no statistically significant differences in bilateral whole hippocampal volume or subfield volumes between the ACMP subgroups with and without cognitive impairment. These findings are presented in Table S2.

After follow up, 37.04% (10/27) ACMP with cognitive impairment developed DEACMP. In contrast, only 15.00% (3/20) patients without cognitive impairment developed DEACMP. Patients with cognitive impairment appeared more likely to develop delayed encephalopathy, although the difference was not statistically significant (*x* ^2^ = 2.789, *p* = 0.095).

**FIGURE 6:**
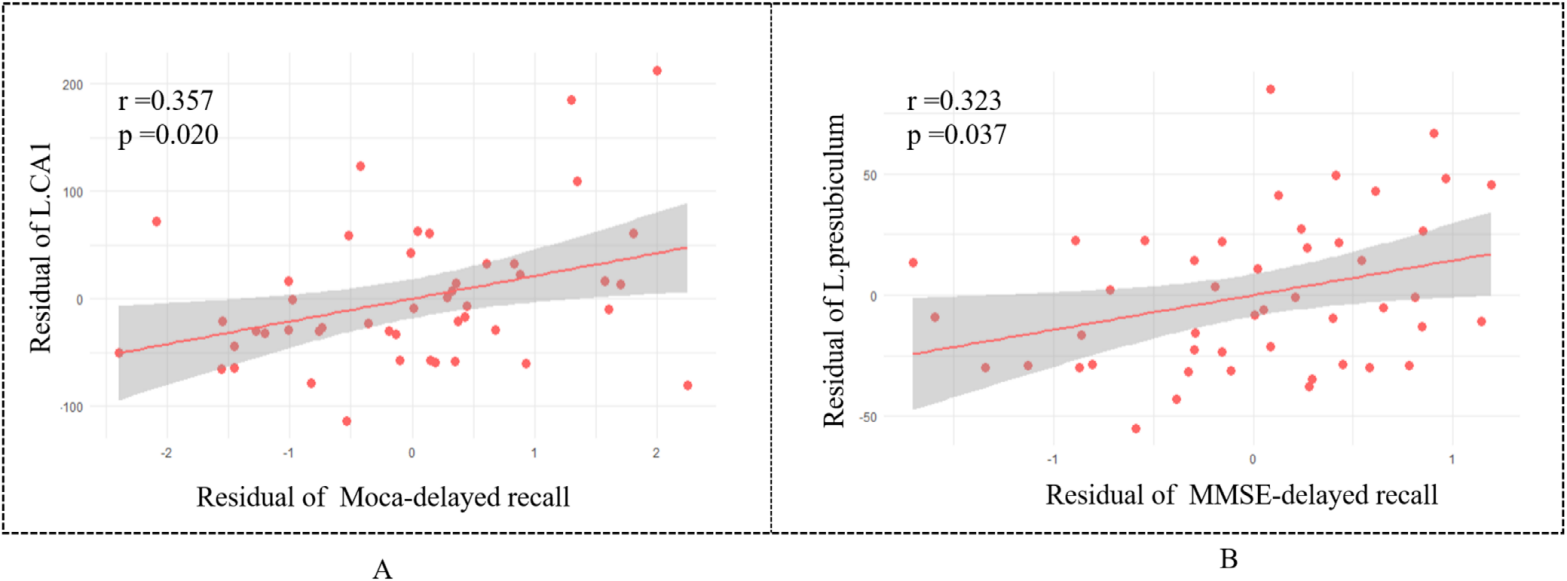
Correlations between hippocampal subfields volume and memory‐related scores. The variables were residuals adjusted for sex, age, education level, ICV, and COHb concentration. ICV, intracranial volume; COHb, carboxyhemoglobin; MoCA, Montreal cognitive assessment; MMSE, mini‐mental state examination.

Correlation analysis showed that MoCA‐delayed recall was positively associated with the volume of the left CA1 subfield (*r* = 0.357, *p* = 0.020), and MMSE‐delayed recall was positively associated with the volume of the left presubiculum (*r* = 0.323, *p* = 0.037) (Figure 6). Besides, no statistically significant correlations were found between hippocampal subfield volumes and the total MoCA or MMSE scores, nor with memory‐related subitems of these assessments.

## 4 Discussion

In the study, we first reported the hippocampal subfield change in the ACMP patients, and also explored the correlations between hippocampal subfield volumes and cognitive function scores. We found ACMP patients have smaller hippocampal volume, mainly located in the bilateral CA3, CA4, GC.ML.DG, Moleculat_layer and right subicum, and DEACMP patients have lower the right subiculum and right CA4 volume compared to the recovery group. Moreover, combined COHB parameters, MoCA and FIM score demonstrated a good predictive value for the occurrence of DEACMP. In addition, we found that over half of the ACMP patients had cognitive impairment, and that memory dysfunction was specifically associated with specific hippocampal subfields. Our results represented a significant step forward in understanding the effects of carbon monoxide poisoning on the hippocampus by revealing the pattern of hippocampal subfield abnormalities, and may not only serve as a non‐invasive and powerful alternative tool for assessing hippocampal damage in patients with ACMP, but also potentially improving a more comprehensive understanding of the neurological impact of CO exposure.

Brain tissue is highly sensitive to hypoxia, with even a few minutes of oxygen deprivation potentially causing significant neurological damage and leading to neuronal death [22, 23]. Carbon monoxide exposure can lead to reduced oxygen delivery to brain tissue, resulting in cellular hypoxia and subsequent neuronal damage [24, 25]. The hippocampus, being a region with high metabolic demands (with a high concentration of mitochondria [26, 27]) and containing densely packed neurons, is particularly susceptible to damage form hypoxia [28, 29, 30]. Previous animal studies showed that the CA3 [31], CA4 [32], DG [33] region was extremely susceptible to ischemia. We found the significant decrease in the volume of the bilateral whole hippocampus in the ACMP group, with the primary affected subfields being bilateral CA3, CA4, GC.ML.DG, Moleculat_layer, and right subiculum. These results not only suggest a pattern of selective vulnerability in hippocampal subfields to ischemic–hypoxic injury but also demonstrate general consistency with the findings from prior animal studies. The observed reductions in the CA3, CA4, GC.ML.DG, and Moleculat_layer subfields may reflect a pattern of neuronal loss and degeneration caused by CO‐induced hypoxia and ischemia. In addition to hippocampal cell damage and loss, inhibited hippocampal neurogenesis may further contribute to hippocampal volume decrease. One research study indicated that ACMP significantly suppresses hippocampal neurogenesis, leading to a reduction in the number of new immature neurons [34]. This may be another reason behind the observed decrease in hippocampal volume.

We found the statistically significant differences between the DEACMP group and the Recovery group, observed in the right subiculum and CA4 subfields with smaller volumes in the DEACMP group. This result suggests a potential link between specific hippocampal subfield atrophy and the development of DEACMP. That's to say, smaller subiculum or CA4 volumes were more likely to be associated with the development of DEACMP. The subiculum and CA4 play critical roles within the hippocampal structure, with the subiculum serving as a major input–output hub [35], connecting the hippocampus to other brain regions, and CA4 extends from CA3 within the curve of the dentate gyrus [36], integral to neurogenesis and memory formation. Given their pivotal roles in these cognitive and emotional pathways, which may be more vulnerable to sustained damage from CO exposure, we speculated that this may be associated with the onset of DEACMP.

However, further ROC curve analysis did not show a significant predictive value for these in identifying DEACMP. This finding suggests that smaller subiculum and CA4 volume were not independently sufficient to reliably predict its occurrence. This could be due to the complex nature of DEACMP's pathophysiology, where the onset and progression, such as the pseudo‐recovery period and reappearance of symptoms during the disease course, may complicate diagnosis, of the disease are influenced by multiple factors beyond hippocampal structure, including individual variability in CO exposure levels, patients' cognitive function, and even other neurobiological factors that may interact with hippocampal damage. Therefore, it would be beneficial for future studies to adopt standardized diagnostic criteria for DEACMP to ensure consistency and comparability with other researches. Besides, a small sample size could be another possible reason.

We found 57.47% ACMP patients had cognitive impairment, which primarily associated with hypoxia‐induced inhibition of mitochondrial respiration and oxidative stress [37, 38], and among patients with cognitive impairment, 37.04% developed DEACMP, while only 15.00% of those without cognitive impairment did. Although the difference was not statistically significant, it suggests a possible trend that needs further confirmation in larger studies. Group comparison results showed that DEACMP patients had poorer cognitive function at admission compared to those in the Recovery group, and cognitive function scores could predict the occurrence of DEACMP. These results suggest that early cognitive impairment after acute CO poisoning might reflect not only immediate brain injury, but also help identify patients who are more likely to develop DEACMP. Moreover, combining COHb levels, which directly reflected the degree of acute CO exposure, with FIM scores, which indicate functional abilities, can further enhance the predictive accuracy.

Correlation analyses revealed that memory‐related scores were associated with specific hippocampal subfields. MoCA‐delayed recall was positively correlated with the volume of the left CA1 subfield (*r* = 0.357, *p* = 0.020), and MMSE‐delayed recall showed a similar association with the left presubiculum (*r* = 0.323, *p* = 0.037). On the one hand, the results suggested that abnormality in the CA1 volume might be mediating changes in cognitive function among patients with ACMP. On the other hand, the positive correlation indicated that a decrease in CA1 volume is associated with poor cognitive status. The CA1 subfield serves as the main output region of the hippocampus and is considered critical for the formation of most memories that rely on hippocampal function [39, 40]. Recent studies have provided evidence suggesting a connection between the volumes of specific hippocampal subfields and verbal episodic memory. For instance, the subiculum has been associated with immediate verbal recall, while delayed recall has been linked to the CA1, subiculum, and presubiculum regions [41, 42]. These findings are consistent with our findings. Based on the aforementioned association between cognitive function and the development of DEACMP, we speculate that the hippocampal specific subfields may be involved in the development of DEACMP by potentially modulating cognitive processes.

There were some limitations in this study. First, out of the 47 patients with ACMP, only 13 were diagnosed with DEACMP, resulting in a relatively small sample size. Future research should aim to include a larger cohort to enhance the robustness of the findings. Second, our study lacked longitudinal follow‐up imaging data, which would provide valuable insights into the temporal progression of hippocampal subfield changes and their correlation with cognitive outcomes over time. Third, the study did not control for potential confounding factors, such as the duration of CO exposure, which may influence both cognitive function and hippocampal structure. Fourth, our study only based on volumetric MRI data, which limits ability to explore hippocampal microstructural integrity, functional connectivity, and metabolic changes. Future studies incorporating multimodal imaging techniques, such as diffusion tensor imaging (DTI), functional MRI (fMRI), and magnetic resonance spectroscopy (MRS), are warranted to provide a more comprehensive assessment of hippocampal pathology in CO poisoning.

In conclusion, we are for the first time to report the pattern of hippocampal injury at the subfields level in patients with ACMP. Bilateral CA3, CA4, GC.ML.DG, Moleculat_layer, and right subiculum were the main affected subfields. The specific hippocampal subfields may be involved in the development of DEACMP, possibly by modulating cognitive functions. Our results may not only contribute to deepening the changes in the hippocampus at the substructural level after ACMP, but also potentially improve understanding of the neurological impact of hypoxic injuries in human subject research.

## Author Contributions

M.T., T.L., and X.Z. conceived and designed the experiments. M.T., T.L., Y.J., N.L., and X.H. performed the experiments. M.T., T.L., and S.W. analyzed the data. M.T., T.L., and X.Z. wrote the manuscript. M.T. and X.Z. reviewed the manuscript.

## Conflicts of Interest

The authors declare no conflicts of interest.

## Supporting information


Data S1.

## Data Availability

The data that support the findings of this study are available from the corresponding author upon reasonable request.
