# Correction to “Abnormalities of Hippocampal Subfields in Individuals With Acute Carbon Monoxide Poisoning”

**DOI:** 10.1111/cns.70556

**Published:** 2025-08-04

M. Tang, T. Li, Y. Deng, et al., “Abnormalities of Hippocampal Subfields in Individuals With Acute Carbon Monoxide Poisoning,” CNS Neuroscience and Therapeutics 31, no. 6 (2025): e70482, https://doi.org/10.1111/cns.70482.

In the originally published version of this article, the authors' affiliation was incomplete. The correct and complete affiliation should be stated as follows: Sichuan Key Laboratory of Medical Imaging, Department of Radiology, Affiliated Hospital of North Sichuan Medical College, Maoyuan South Street 1, Nanchong 637000, Sichuan, PR China.

We apologize for this error.

