# Supplementary material for: Abnormalities of Hippocampal Subfields in Individuals With Acute Carbon Monoxide Poisoning
Source: CNS Neurosci Ther. 2025 Jun 23;31(6):e70482. doi: 10.1111/cns.70482 (PMC12183523; doi:10.1111/cns.70482)
Supplement: Supplementary file 1 — Data S1. [file CNS-31-e70482-s001.docx]

**Supplementary**

**Figure 1**


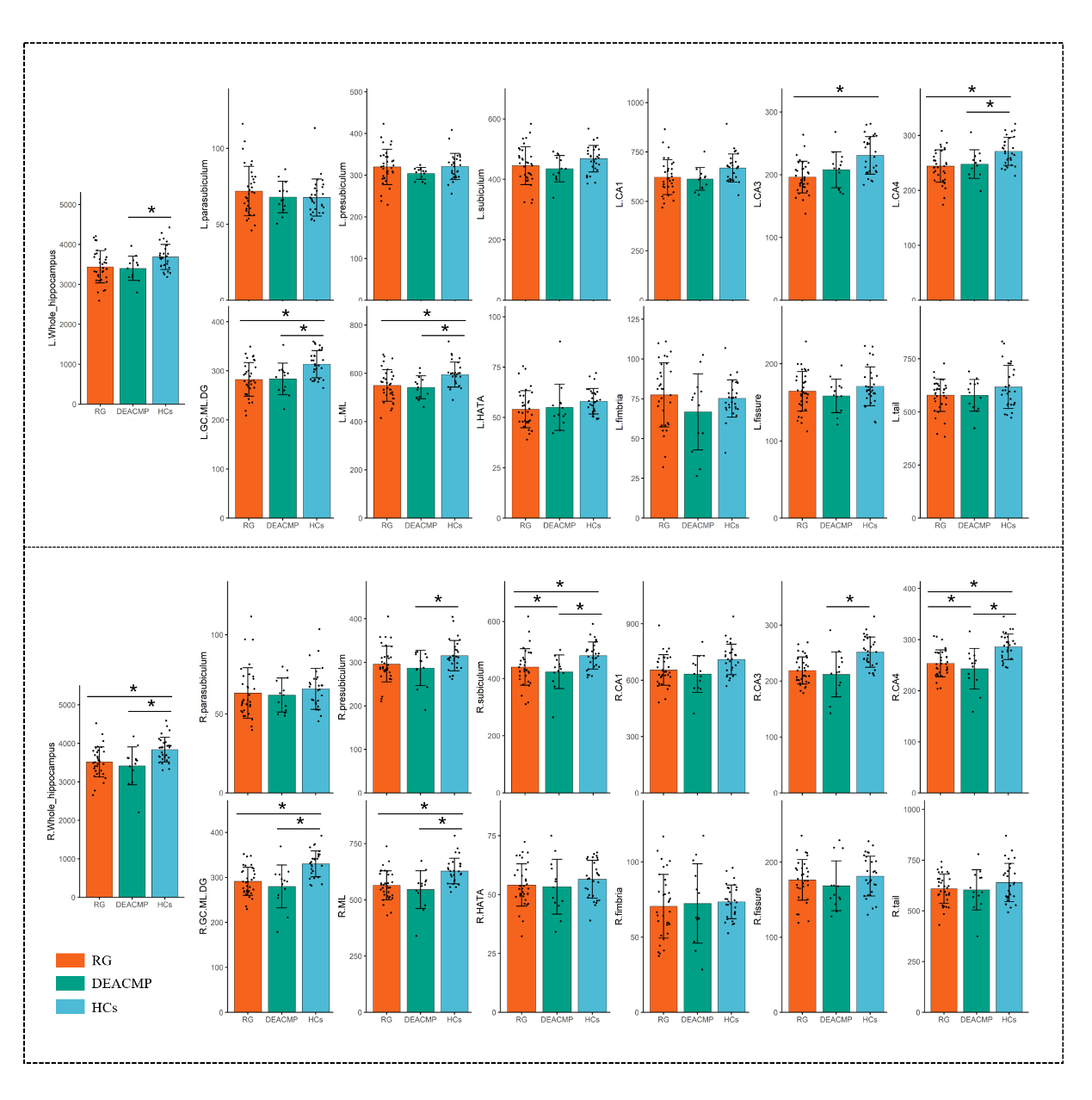


**Figure legends**

**Figure 1** Comparison of hippocampal subfields volume among the DEACMP, RG and HCs. * indicates a statistically significant difference after FDR correction. (p.FDR ＜0.05). RG, Recovery group; DEACMP, Delayed Encephalopathy After Carbon Monoxide Poisoning; HCs, healthy controls; CA, cornu ammonis; GC.ML.DG, Granule cell and molecular layer of the dentate gyrus; ML, molecular _layer; HATA, Hippocampus-amygdala transition area.

**Figure 2**


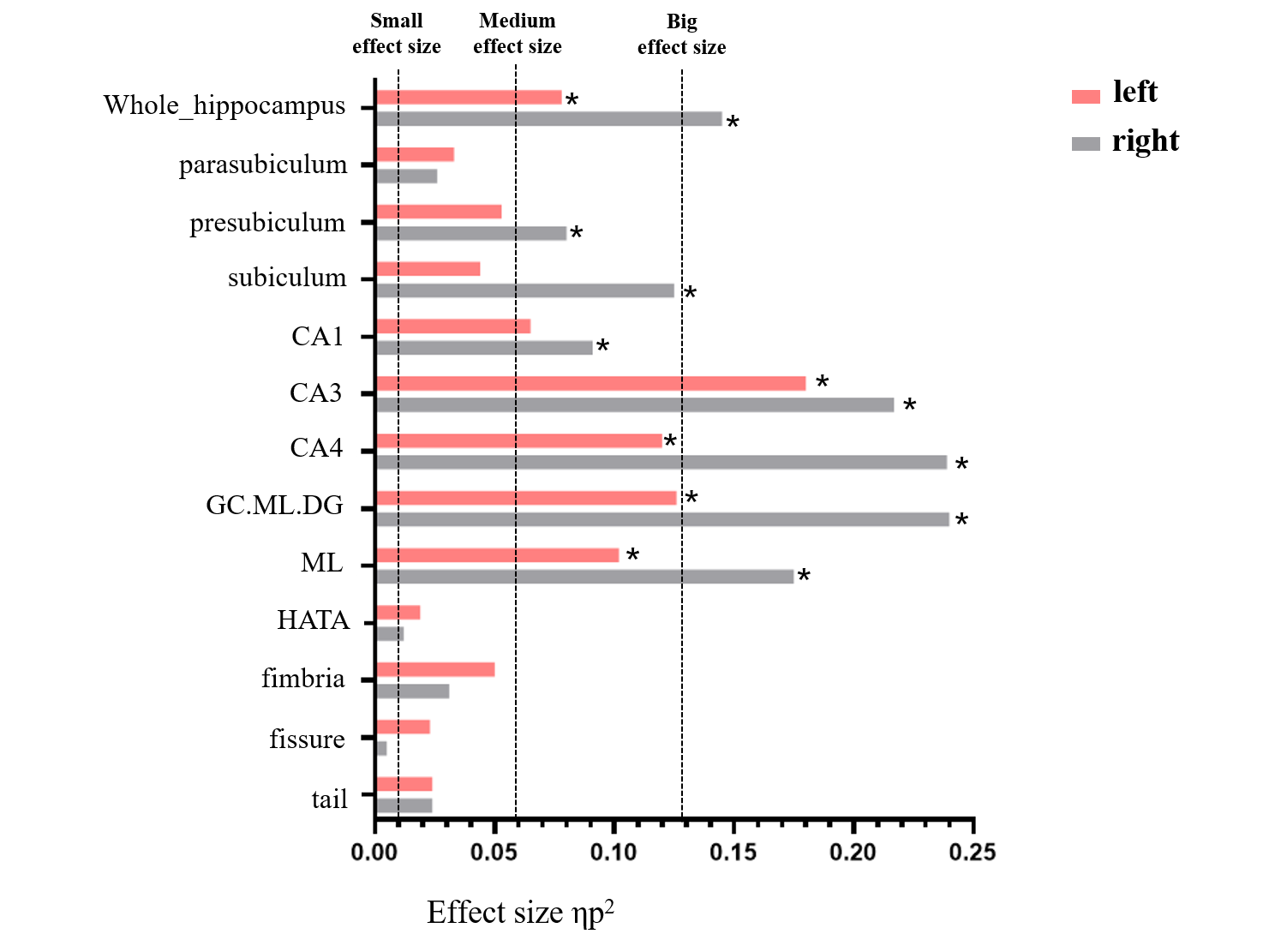


**Figure legends**

**Figure 2** Effect size of bilateral whole hippocampus and hippocampal subfields among among the RG, DEACMP and HCs. * indicates a statistically significant difference after FDR correction. (p.FDR ＜0.05). RG, Recovery group; DEACMP, Delayed Encephalopathy After Carbon Monoxide Poisoning; HCs, healthy controls; CA, cornu ammonis; GC.ML.DG, Granule cell and molecular layer of the dentate gyrus; ML, molecular _layer; HATA, Hippocampus-amygdala transition area.

**Table 1 ROC curve analysis results**

|  | AUC | SE | P value | 95% CI | |
| --- | --- | --- | --- | --- | --- |
|  |  |  |  | lower | upper |
| COHb | 0.712 | 0.078 | 0.026 | 0.560 | 0.863 |
| MoCA-delayed recall | 0.730 | 0.089 | 0.016 | 0.556 | 0.904 |
| MoCA-delayed recall | 0.696 | 0.097 | 0.040 | 0.505 | 0.887 |
| MMSE | 0.717 | 0.083 | 0.022 | 0.554 | 0.881 |
| MMSE-delayed recall | 0.785 | 0.075 | 0.003 | 0.638 | 0.932 |
| FIM | 0.801 | 0.082 | 0.002 | 0.639 | 0.962 |
| ADL | 0.701 | 0.097 | 0.034 | 0.512 | 0.891 |

Note: AUC, Area Under the ROC Curve; COHb, carboxyhemoglobin; MoCA, montreal cognitive assessment; MMSE, Mini-Mental state examination; FIM, functional independence measurement; ADL, activities of daily living.

**Figure 3**


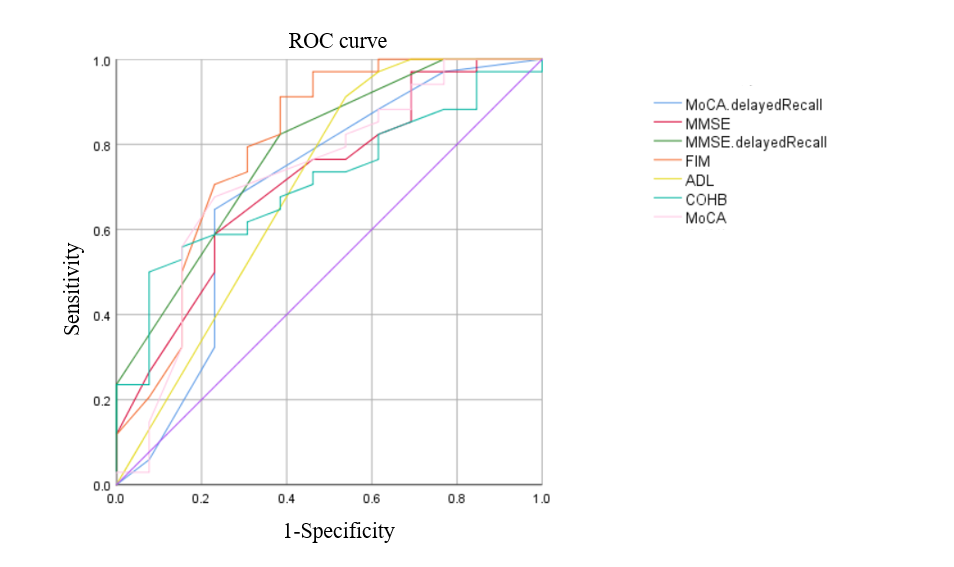


**Figure legends**

**Figure 3** ROC curve analysis to evaluate the prediction value for DEACMP. ROC, Receiver operating characteristic; DEACMP, Delayed Encephalopathy After Carbon Monoxide Poisoning; COHb, carboxyhemoglobin; MoCA, montreal cognitive assessment; FIM, Functional Independence Measurement.

**Table 2 Hippocampal Volume Comparison in ACMP subgroups with and without cognitive impairment**

|  | without cognitive impairment （N=27） | |  | with cognitive impairment （N=20） | | F value | p value | effect size |
| --- | --- | --- | --- | --- | --- | --- | --- | --- |
|  | Mean | SD |  | Mean | SD |  |  |  |
| L.Whole_hippocampus | 3453.631 | 355.594 |  | 3400.765 | 410.833 | 0.229 | 0.635 | 0.006 |
| L.parasubiculum | 72.384 | 13.788 |  | 68.648 | 16.318 | 0.437 | 0.512 | 0.011 |
| L.presubiculum | 318.579 | 36.875 |  | 311.324 | 38.308 | 0.462 | 0.501 | 0.011 |
| L.subiculum | 450.036 | 59.908 |  | 431.800 | 54.060 | 0.002 | 0.964 | 0.000 |
| L.CA1 | 624.352 | 66.775 |  | 614.288 | 97.428 | 0.443 | 0.509 | 0.011 |
| L.CA3 | 200.979 | 26.826 |  | 197.526 | 26.518 | 0.000 | 0.991 | 0.000 |
| L.CA4 | 247.869 | 25.906 |  | 241.748 | 31.515 | 0.021 | 0.885 | 0.001 |
| L.GC.ML.DG | 285.219 | 30.818 |  | 279.594 | 36.961 | 0.039 | 0.845 | 0.001 |
| L.ML | 551.975 | 55.447 |  | 541.935 | 67.986 | 0.096 | 0.759 | 0.002 |
| L.HATA | 54.058 | 9.902 |  | 54.711 | 9.833 | 0.293 | 0.591 | 0.007 |
| L.fimbria | 69.848 | 20.926 |  | 80.860 | 21.264 | 1.777 | 0.190 | 0.042 |
| L.fissure | 161.981 | 24.289 |  | 164.061 | 25.472 | 1.796 | 0.188 | 0.042 |
| L.tail | 578.333 | 76.683 |  | 578.332 | 74.613 | 0.044 | 0.835 | 0.001 |
| R.Whole_hippocampus | 3498.836 | 475.449 |  | 3473.942 | 337.931 | 0.499 | 0.484 | 0.012 |
| R.parasubiculum | 65.835 | 16.502 |  | 58.734 | 10.694 | 0.097 | 0.757 | 0.002 |
| R.presubiculum | 297.507 | 41.925 |  | 288.082 | 39.203 | 0.012 | 0.914 | 0.000 |
| R.subiculum | 439.458 | 68.339 |  | 431.222 | 54.577 | 0.515 | 0.477 | 0.012 |
| R.CA1 | 645.052 | 94.771 |  | 652.439 | 74.560 | 1.108 | 0.299 | 0.026 |
| R.CA3 | 215.596 | 32.864 |  | 218.770 | 24.113 | 1.090 | 0.303 | 0.026 |
| R.CA4 | 251.509 | 35.939 |  | 249.342 | 22.370 | 0.272 | 0.605 | 0.007 |
| R.GC.ML.DG | 288.568 | 42.760 |  | 287.541 | 26.454 | 0.290 | 0.593 | 0.007 |
| R.ML | 558.472 | 80.066 |  | 560.774 | 54.887 | 0.618 | 0.436 | 0.015 |
| R.HATA | 52.394 | 9.328 |  | 55.842 | 9.960 | 3.992 | 0.052 | 0.089 |
| R.fissure | 173.838 | 28.157 |  | 174.703 | 30.208 | 2.621 | 0.113 | 0.060 |
| R.fimbria | 69.479 | 23.634 |  | 73.154 | 21.130 | 0.003 | 0.954 | 0.000 |
| R.tail | 614.967 | 90.467 |  | 598.041 | 62.774 | 0.001 | 0.980 | 0.000 |

Note: ACMP, After Carbon Monoxide Poisoning
